# 

**Published:** 1886-07

**Authors:** 


### INDEX.

### A.

PAGE.

Accoucheur—How soon after exposure to sepsis may he resume practice, . . . . . . . . . . 31
Antipyrine in sunstroke, . . . . .
Agoraphobia, . . . . . . . . . . 90
Address, Introductory. Thos. Lothrop, M. D., . . . . . . . . . . . 145
Asthma, treatment of . . . . . . 220
Abortion. How shall we manage 241
American Public Health Ass'n, . 286
Army Surgeons, The. F. H. Hamilton, M. D., . . . . . . . . . . 297
Agnew, D. Hayes, M. D. Painful Muscular Spasm, . . . . . . . 325
Asylum, Providence . . . . . . 335
Angell, E. B., M. D., Reflex Action its Value in the Diagnosis of Spinal Diseases, . . . . . . . . . . . . 345
Address to Graduating Class, Niagara University, Simeon T. Clark, M. D., . . . . . . . . . . 443
Amenorrhœa, Dr. Fordyce Barker on treatment of . . . . . . . 466

### B.

Buffalo Medical and Surgical Association, 9, 78, 111, 171, 215, 357, 411
Buffalo Obstetrical Society, . . . . . . . . . . . . . . 13, 256, 406, 459
Buffalo Hospitals, Editorial on . . 39
Bronchiectasis, John O. Roe, M. D., 97
Buffalo Colleges. Editorial, . . . . 143
Buffalo Medical and Surgical Association. Editorial, . . . . . . 136

PAGE

Buffalo Medical Library Association, 275
Bovine Vaccine Virus, . . . . . . 287
Banta, R.L., M.D.,Placenta Prævia, 339
Buffalo Maternity Hospital, . . . . 492
Butter, Fats, Microscopical Examination of, George E. Fell, M. D., . 510

### C.

Cholera Infantum, Morphia in . . 32
County Medical Societies, Membership of . . . . . . . . . . 38
Correspondence,Foreign, . . . . . 68
Correspondence, London Hospitals, etc, C. C. F. Gay, M. D., . . . 172
Correspondence, Dr. Bigelow, from Berlin, . . . . . . . . . . . . . 122
Chloral Hydrate, External use of . 88
Castor Oil, palatable, . . . . . . 416
Correspondence from William J. Cronyn, M. D., . . . . . . . . . 129
Correspondence from M. Hartwig, M. D , . . . . . . . . . . . . . 133
"Preposition," . . . . . . . . . 278
Chloral Hydrate Bromide of Potassium, Incompatibility of . . . . 139
Campbell, F. R., M. D., Relation of Meteorology to Disease, . . . . 193
Cocaine Habit, The . . . . . . . 225
Cholera, . . . . . . . . . . . . 274
Cocaine in Whooping Cough, . . . 374
Cremation, Editorial, . . . . . . 333
Cocaine, Opacity of Cornea Produced by . . . . . . . . . . . 376
College Commencement, Buffalo Medical, . . . . . . . . . . . . 378

PAGE.
Clark, Simeon T., M. D., Address, 443
Chancre, Hebra's Treatment of, . 581

D.

Davidson, A. R., M. D., Diabetes Mellitus, . . . . . . . . . . . . 49
Davidson, A. R., M. D., Differential Diagnosis of Gastric Ulcer, Chronic Gastritis and Gastric Cancer, . . 495
Davidson, A. R., M. D., Intubation of Larynx, . . . . . . . . . . 553
Diabetes Mellitus, A. R. Davidson, M. D, . . . . . . . . . . . . . 49
Disinfection of Physicians' Hands, 89
Diphtheria, a New Method of Treating . . . . . . . . . . . . 268
Dysentery, Etiology of . . . . . 579

E.

Esmarch, Transplanting Skin, . . 20
Emmett's Operation, . . . . . . . 29
Endometritis, Purulent during Pregnancy, . . . . . . . . . . 31
Enuresis, Rhus Toxicodendron in treatment of . . . . . . . . . 90
Epithelioma of Cervix, Complicating Labor, C. C. Frederick, M.D., 155
Erie County Medical Society Proceedings, . . . . . . . . . . 171, 304
Elephantiasis of Hand, B. P. Hoyer, M. D., . . . . . . . . . . . . . 452
Earth, Topical Use of . . . . . . 467

F.

Fallopian Tubes, Diseases of . . . 24
Fucus Marina, B. Rosmer, M. D., . 32
Frederick, C. C., M. D. Epithelioma of Cervix, Complicating Labor, . 155
Fractures, Treatment of Non-Union of . . . . . . . . . . . . . . . 376
Flint, Austin, M. D., Professor. Editorial, . . . . . . . . . . . . . 429
Food, Infants, English and American, . . . . . . . . . . . 472
Fell, George E., M. D. Microscopic Examination of Butter and other Fats, . . . . . . . . . . . . . . 510
Fats, Absorbability of, by the Skin, 521

G.

Gonorrhœa in the Female, Diagnosis of . . . . . . . . . . . . . . . 88
Glycosuria, Temporary in Surgical Affections, . . . . . . . . . . . 126
Gay, C. C. F., M. D. Letters from London, . . . . . . . . . . 68, 172
Germ Theories, Inconsistences of . 265
Gynæcology, Overgrowth of Surgery in . . . . . . . . . . . . . . . 477
Gay, Charles Curtis Fenn. Editorial, 482
Gastric Cancer, Ulcer and Gastritis, A. R. Davidson, M. D., . . . . 495
Gonorrhœa, Treatment of Chronic, by Grooved Sounds, . . . . . . 532

H.

Hysterectomy, Vaginal, for Cancer, 35
Hayd, Herman E., M. D., . . . . 106
Hemorrhoids, Treatment by Injections, . . . . . . . . . . . . . 126
Hydrophobia and Pasteur's Experiments. Editorial, . . . . . . . 226
Hartwig, M., M. D. How shall we Manage Abortion, . . . . . . . 241
Hamilton, Frank H., M. D. The Army Surgeons, . . . . . . . . . 297
Hypnotism during Parturitions, . . 324
Hiccough, A New Remedy Food, . 328
Hypnotics, Dujardin-Beaumetz on 273
Hemorrhage, Theophilus Parvin, M. D., . . . . . . . . . . . . . . 387
Hoyer, B. P. Elephantiasis of Hand, 452
Hospital, Buffalo Maternity, . . . 492

I.

Intestinal Helminthiaisis and Disorders of Vision, . . . . . . . . 73
Impotence, Treatment of Ultzman, 81
Incontinence of Urine, Treatment of 89
Intra-cranial Cephalhæmatoma, . . 272

PAGE.
Insanity, Alcoholic. Editorial, . . 281
Intro-Cranial Tumor, Extirpation of 369
Ingraham, Henry D., M. D. Removal of Ovarian Tumor, . . . 397
Intubation of Glottis, A. R. Davidson, M. D., . . . . . . . . . . 495

### L.

Lithæmia. J. W. Putnam, M. D., 1
Lothrop, Thomas, M. D. Address to Students, . . . . . . . . . 145
Lung, Surgery of . . . . . . . . 273
Laparotomy Explorative. G. R. Fowler, M. D., . . . . . . . . . 316
Lithæmia, Hippurate Soda in . . . 419
Lanoline, . . . . . . . . . . . . 436
Larynx, Extirpation of the . . . 479
Larynx, Intubation of. A. R. Davidson, M. D., . . . . . . . . . 553

### M.

Menstruation, Vicarious, . . . . . 581
Medical News—Items, . . 127, 181, 329
Mickle, H. H., M. D. Notes of Cases at Emergency Hospital, . . 165
Meteorology to Disease, Relations of. F. R. Campbell, M. D., . . . 193
Melancholia, Treatment of . . . 225
Midwives. List of Licensed in Erie County, . . . . . . . . . . . 277
Meningitic Affections, Peculiar Phenomenon in . . . . . . . . . . 319
Muscular Spasm—Excision of Portion of Nerve Trunk, . . . . . 325
Menthol, in Treatment of Pruritus, 382
McPherson, Dr. Donald F. Edit'l, 439
Medical Legislation. Editorial, . 533

### N.

Nasal Septum, Deviation of the . 86
Night Calls, to Avoid . . . . . . 126
Nasal Stenosis, . . . . . . . . . . 270
Nutrition in Lithæmia. Dr. C. G. Stockton, . . . . . . . . . . . 291
Niagara University, First Convocation of Medical Department, . . 486
Nerves, Suture of . . . . . . . . 578

### O.

Oxytocic, Heat as an . . . . . . 89
Obituary, Dr. A. B. Parsons, . . 280
Obituary, Austin Flint, M. D., LL. D. 425
Ovarian Tumor, Removal of. H. D. Ingraham, M. D., . . . . . . . 397

### P.

Pregnancy, Early Diagnosis of. E. S. McKee, M. D., . . . . . . . 563
Providence Asylum for the Insane, 537
Putnam, J. W., M. D. Lithæmia, 1
Proceedings Buffalo Med. and Surg. Asso'n, . . . . . . . . . . .
. 7, 78, 111, 171, 215, 357, 411, 575
Pereirine, in Malarial Fevers, . . 528
Proceedings Buffalo Obstetrical Society, . . . . . . . . . 13, 406, 459
Proceedings Erie Co. Society, 171, 304
Proceedings Rochester Pathological Society, . . . . . . . . . 218, 360
Proceedings Central New York Medical Society, . . . . . . . . . 567
Parvin, Prof. Clinical Lectures, . 387
Prize for Best Essay. Med. Society State of New York, . . . . . . 38
Plastic Splints in Treatment of Fractures, . . . . . . . . . . . . 82
Pneumonia-coccus of Friedlander, 112
Phthisis. Treatment of by Antiseptic Inhalation, . . . . . . . . . 176
Phthisis. Treatment by Sub-cutaneous Administration of Carbolic Acid, . . . . . . . . . . . . 323
Parturition. Hypnotism during . 324
Pruritus Vulvæ. Surgical Treatment of . . . . . . . . . . . 326
Placenta Prævia, Treatment of. R. L. Banta, M. D., . . . . . . . . 339
Ptomaines and Leucomaines, . . . 366
Peptonuria, Puerperal . . . . . 478
Pichi, a New Remedy in Cystitis, . 526

PAGE.

Potter, W. W., M. D. Some Observations on the Uterine Sound, . 544

### R.

Roe, John O., M.D. Bronchiectasis, 97
Rochester Pathological Society, 218, 360
Rhino-Lithiasis. Dr. O. Chiari, . 311
Rochester, Thos. F. Editorial, . 381
Renal Hemorrhage. Wm. G. Ring, M. D., . . . . . . . . . . . 404
Rabies, The Prevention of. Editorial, . . . . . . . . . . . . . 434

### S.

Syphilis, Hypodermic Medication in 87
Stricture Urethral. H. E. Hayd, M. D., . . . . . . . . . . . . . 106
Sclerosis, Disseminated Cerebro Spinal, . . . . . . . . . . . . 114
Sulphide of Potassium, Lotions of 124
Small-pox, Inspection ag ainst . 182
Stockton, Chas. G., M. D. Nutrition in Lithæmia, . . . . . . . 291
Spinal Diseases and Reflex Action. E. B. Angell, M. D., . . . . . 345
Sterility, Treatment of . . . . . 580

### T.

Transplanting Large Portions of Skin on Fresh Wounds. Esmarch, 20
Tyrotoxicon—Cheese Poison, . . . 26

PAGE.

Thyroid Gland, Functions of . . 121
Tait, Lawson, on Surgery of Kidney, 222
Tobacco, Physiological Action of 322
Thalline, a New Antipyretic, . . . 475
Training School for Insane Asylum Attendants. First Annual Commencement, . . . . . . . . . . 491
Tremaine, W. S., M. D., Supra-Pubic vs. Perineal Lithotomy, . . . . 522

### U.

Urethane. Editorial, . . . . . . 582
Urethrane, or Carbonate of Ethyl, 413
Uterine Disease, Dry Treatment of 472
Urethane, Physiological Action of 533
Uterine Sound, Some Observations on. W. W. Potter, M. D., . . . . 544

### V.

Vaughan, V. C. Tyrotoxicon, . . 26
Volume xxv. Editorial, . . . . 36

### W.

Whooping Cough, Cocaine in . . 328
Warts, Isquierdo on . . . . . . 468
Water, Micro-Organisms of Potable, 480

### Y.

Yellow Fever, Preventive Inoculation for . . . . . . . . . . . 529

